# Integrated Assessment of Antibacterial Activity, Polyphenol Composition, Molecular Docking, and ADME Properties of Romanian Oak and Fir Honeydew Honeys

**DOI:** 10.3390/antibiotics14060592

**Published:** 2025-06-08

**Authors:** Calin Hulea, Diana Obistioiu, Anca Hulea, Mukhtar Adeiza Suleiman, Doris Floares (Oarga), Ersilia Alexa, Ilinca Merima Imbrea, Alina-Georgeta Neacșu, Marius Pentea, Cosmin Alin Popescu, Florin Imbrea

**Affiliations:** 1 Faculty of Veterinary Medicine, University of Life Sciences “King Michael I” from Timisoara, Calea Aradului 119, 300645 Timisoara, Romania; calin.hulea@usvt.ro (C.H.); mariuspentea@usvt.ro (M.P.); 2 Faculty of Agriculture, University of Life Sciences “King Michael I” from Timisoara, Calea Aradului 119, 300645 Timisoara, Romania; anca.hulea@usvt.ro (A.H.); doris.oarga@usvt.ro (D.F.); alinaneacsu@usvt.ro (A.-G.N.); cosmin_popescu@usvt.ro (C.A.P.); florin_imbrea@usvt.ro (F.I.); 3 Faculty of Life Science, Department of Biochemistry, Ahmadu Bello University, Zaria 810107, Nigeria; masuleiman@abu.edu.ng; 4 Faculty of Food Engineering, University of Life Sciences “King Michael I” from Timisoara, Calea Aradului 119, 300645 Timisoara, Romania; ersiliaalexa@usvt.ro; 5 Faculty of Engineering and Applied Technologies, University of Life Sciences “King Michael I” from Timisoara, Calea Aradului 119, 300645 Timisoara, Romania; ilinca_imbrea@usvt.ro

**Keywords:** honeydew honey, oak honey, fir honey, polyphenols, antibacterial activity, antioxidant activity, molecular docking, ADME properties

## Abstract

**Background:** This study evaluated the polyphenolic composition, antibacterial activity, molecular docking interactions, and pharmacokinetic properties of Romanian oak and fir honeydew honeys. **Methods:** Spectrophotometric methods quantified total phenolic, flavonoid contents and antioxidant activity, and individual polyphenols were identified via HPLC-MS. Antibacterial efficacy against Gram-positive and Gram-negative bacteria was evaluated by determining the bacterial inhibition percentage and minimum inhibitory concentrations. The bioactive compounds identified via LC-MS analysis were used to further delineate the possible antibacterial activities in silico. Molecular docking was carried out to predict the binding interactions and complex formation of the identified compounds against protein crystal structures of the bacteria used in this study. Additionally, the pharmacokinetic profile of compounds with high inhibitory potential was assessed via ADMET (absorption, Distribution, Metabolism, Excretion, toxicity) predictors to ascertain their value. **Results:** Fir honeydew honey showed higher total phenolic (844.5 mg GAE/kg) and flavonoid contents (489.01 mg QUE/kg) compared to oak honeydew honey, correlating with more potent antioxidant activity (IC50 = 5.16 mg/mL). In vitro antimicrobial tests indicated a stronger inhibitory effect of fir honeydew honey, especially against Gram-positive strains like *S. aureus*, *S. pyogenes*, and *L. monocytogenes*, alongside certain Gram-negative strains such as *E. coli* and *H. influenzae*. Oak honeydew honey displayed selective antimicrobial action, particularly against *P. aeruginosa* and *S. typhimurium*. The docking outcomes showed rutin, rosmarinic acid, beta resorcylic acid, quercetin, ferulic acid, and p-coumaric acid have high inhibitory activities characterised by binding affinities and binding interactions against shiga toxin, riboflavin synthase, ATP-binding sugar transporter-like protein, undecaprenyl diphosphate synthase, putative lipoprotein, sortase A, and immunity protein, making them key contributors to the honey’s antimicrobial activity. Moreover, beta-resorcylic acid, quercetin, ferulic acid, and p-coumaric acid revealed interesting ADMET scores that qualify honey to serve as a good antimicrobial agent. **Conclusions:** These findings support their potential use as natural antibacterial agents and emphasise the value of integrating chemical, biological, and computational approaches for multidisciplinary characterisations.

## 1. Introduction

Honey, a naturally sweet substance made by *Apis mellifera*, is among the earliest traditional remedies valued for its ability to treat various ailments, owing to its anti-inflammatory [1,2], antimicrobial [2,3,4], antifungal [5], and antiviral properties [6]. The nutritional and biological properties of honey stem from its various components, including sugars, proteins, free amino acids, organic acids, vitamins, minerals, enzymes, flavonoids, and phenolic acids [7,8,9,10,11,12,13,14]. Several factors influence the levels of these compounds, including geographical origin, botanical source, climate and weather conditions, treatment methods, and importantly, the conditions under which they are harvested, processed, and stored [15,16,17]. The differences in the biological and health-promoting properties of various honeys can be attributed to the varying levels of phytochemical and bee-derived compounds, which are influenced by these factors [18,19,20].

Among the biological characteristics, honey exhibits strong antioxidant activity, being capable of preventing damage caused by oxidants such as OH−, O_2_, superoxide, and lipid peroxyl radicals. However, the values of antiradical activity, a metric used to quantify honey’s antioxidant qualities, obtained by different methods, are variable depending on various factors, especially floral origin [21,22]. The plants from the Rosaceae, Fabaceae, Asteraceae, and Amaranthaceae families positively impact the quantity of antioxidants in honey [22]. On the other hand, dark honey, such as buckwheat, thyme, dandelion, wildflower, chestnut, meadow, manna, and manuka honey, is characterised by higher antioxidant activity than light honey due to a higher content of polyphenolic compounds [23,24,25]. Honey contains various flavonoids and phenolic compounds, including kaempferol, chrysin, quercetin, pinobanksin, luteolin, pinocembrin, apigenin, naringenin, hesperetin, genistein, p-coumaric acid, ferulic acid, gallic acid, syringic acid, vanillic acid, and caffeic acid [26,27,28,29]. The exact way these compounds affect oxidative stress—by either reducing or inhibiting it—is not completely understood. Nonetheless, it is thought to involve free radical sequestration, metallic ion chelation, hydrogen donation, and the action of flavonoid substrates on hydroxyl and superoxide radicals [21,30].

Besides its antioxidant activity, honey is recognised as an antimicrobial agent due to its high osmolarity, low pH, H_2_O_2_ content, and non-peroxide compounds such as bee-defensin one and flavonoids, respectively, and phenolic acid levels [3,12,31,32,33,34]. The mechanism of action has not been fully elucidated. Still, it has been demonstrated that the bactericidal effect is due to membrane permeability alteration, with potassium and protein leakage, inhibition of membrane and intracellular protein synthesis, and bacterial DNA damage [35]. Over time, different types of honey have demonstrated their antibacterial efficacy, both against Gram-positive and Gram-negative bacteria such as methicillin-resistant *Staphylococcus aureus* (MRSA), *Streptococcus pneumoniae*, *Bacillus subtilis*, *Listeria monocytogens*, *Escherichia coli*, *Pseudomonas aeruginosa*, *Salmonella* spp., *Klebsiella pneuomniae* [26,32,35,36,37,38]. The values of minimum inhibitory concentrations for each bacterial strain vary from one type of honey to another in different regions of the world, being, in general, lower for Gram-positive than Gram-negative bacteria [26,37,38]. The different cell wall compositions can explain the difference in susceptibility to honey between the two types of bacteria. An outer membrane protecting the peptidoglycan layer characterises Gram-negative bacteria, while Gram-positive bacteria are surrounded only by a thick peptidoglycan layer [39]. Still, several investigations indicated that Gram-positive bacteria are more resistant to various types of honey than Gram-negative bacteria, most likely due to the samples’ increased hydrogen peroxide content and osmolality [40,41]. Although the minimum inhibitory concentrations observed in the literature are variable, depending on the type of honey and its physicochemical characteristics, as well as on the bacterial strains studied, one thing is certain: unlike synthetic antibiotics, microbial resistance to honey has never been reported [42]. This aspect is critical since the increasing emergence of multidrug-resistant bacteria in recent decades represents a significant challenge worldwide for veterinary and public health [21,43,44,45]. However, the biological activity of honey is influenced by the bioavailability of different phytochemical components as well as how they are absorbed and metabolised [46].

Romanian honey production is notable for its high quality and wide variety due to the temperate-continental climate of the country and melliferous plants found across the Carpathian-Danubian-Pontic area [47]. Moreover, it is primarily produced in rural areas, where beekeepers maintain hives in the heart of the countryside, away from industrial pollutants. This aspect contributes to its purity and minimal contamination [48,49]. The diverse relief, different climatic characteristics, and the variable vegetation from one region to another influence honey production and its biological properties throughout the country. The most common types of honey produced in all areas are rapeseed honey, acacia honey, linden honey and polyfloral honey. In addition, honeydew honey—a non-floral honey—is made in mountainous regions of coniferous and deciduous forests. Unlike other kinds of honey, this type is obtained from manna, the sweet secretions produced by aphids and scale insects, which feed on the sap of plants and trees [50]. Few studies have characterised honeydew honey, although it is claimed that the antioxidant and antimicrobial activities are superior to other types of monofloral honey [51], except for manuka honey. Furthermore, to our knowledge, there are no data regarding the biological activity of the varieties of honeydew honey in Romania.

Computer-based virtual screening on the key proteins of the microorganisms has led to the identification and understanding of the mechanism of action by which the inhibition of compounds could match the known ligands of the proteins. Notably, specific compounds responsible for the antimicrobial activities possess a control selectivity tied to the reactive groups’ physicochemical functionality [52]. With the continuous surge in antimicrobial resistance to the available antibiotics, new antibacterial agents from natural origin, which contain bioactive molecules targeting necessary metabolic enzymes of microbial pathogenesis, could be assessed in silico. Thus, the crystal structure of seven bacterial protein targets, shiga toxin, riboflavin synthase, ATP-binding sugar transporter-like protein, undecaprenyl diphosphate synthase, putative lipoprotein, sortase A, and immunity protein are suitable targets for antimicrobial assessment due to their key involvement in the machinery of protein synthesis and bacterial growth ranging from metabolic enzymes, cell wall, virulence factors and key genes necessary for microbial pathogenicity [53].

The samples analysed were sourced from approved local producers from Sibiu, Romania. The Sibiu region in the Southern Carpathians is characterised by extensive mixed forests dominated by fir (*Abies alba*) and oak (*Quercus* spp.), providing ideal conditions for honeydew secretion. This area is known for its high-quality honeydew honey, which is traditionally valued for its rich mineral content and distinctive dark colour.

Although various studies have tested the bioactive potential of different monofloral honeys, there is little information available regarding honeydew honeys of different botanical origins, especially fir (FHD) and oak (OHD). Additionally, there have not been many studies linking the polyphenolic profiles of these honeys to their respective quantitative antimicrobial efficacies against a variety of clinically relevant bacterial strains. This study bridges this gap by providing a detailed comparative analysis of the chemical constituents and antimicrobial performance of FHD and OHD using LC-MS phenolic profiling, quantitative antimicrobial metrics (BIP%, MIC) and molecular docking. The main objectives of the research were as follows: (i) to determine the total phenolic (TPC), flavonoid content (TFC) and antioxidant activity; (ii) identify and quantify the major polyphenolic compounds in FHD and OHD using HPLC-MS; (iii) to evaluate the antibacterial activity of OHD and FHD against a panel of clinically relevant Gram-positive and Gram-negative ATCC bacterial strains; (iv) to perform molecular docking simulations of selected polyphenols (e.g., ferulic acid, caffeic acid, resveratrol, quercetin) against key bacterial target proteins such as DNA gyrase and penicillin-binding proteins; (v) to evaluate the ADME properties (absorption, Distribution, Metabolism, and Excretion) of the identified bioactive compounds using in silico pharmacokinetic models. To correlate the antibacterial efficacy with the polyphenolic profile and computational results, identifying which compounds contribute most to antimicrobial potential.

## 2. Results

### 2.1. Determination of Total Phenolic Content (TPC) and Flavonoid Content (TFC)

**Figure 1:**
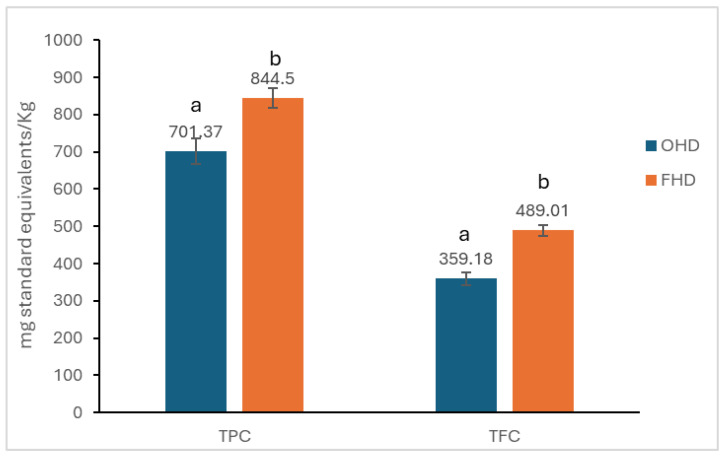
TPC and TFC of honey samples. The mean of the three determinations ± standard deviation (SD) is used to express the results. Different lowercase letters (a, b) indicate statistically significant differences between samples from TPC and TFC according to one-way ANOVA followed by Tukey’s HSD test (*p* < 0.05).

The results for total phenolic content (TPC) and flavonoid content (TFC) are presented in Figure 1.

The total polyphenol content in the ODH sample was 701.37 mg GAE/Kg, while in the FHD sample, the content was 844.5 mg GAE/Kg. There were statistically significant differences (*p* < 0.05) in the total polyphenol content between the two samples of analysed honey.

The TFC in the ODH sample was 359.18 mg QUE/Kg, while in the FHD sample, the content was 489.01 mg QUE/Kg. The two samples have statistically significant differences (*p* < 0.05).

### 2.2. Antioxidant Capacity by 1,1-Diphenyl-2-picrylhydrazyl (DPPH) Assay

The results of the antioxidant activity determined by the DPPH assay are presented in Table 1.

The highest inhibition is observed at the highest studied concentration (100 mg/mL) for both samples. The FHD sample demonstrates a greater radical scavenging activity (52.51%) than the OHD sample (42.93%). Statistically significant differences (*p* < 0.05) are noted at nearly all concentrations between the two samples, except for the 25 mg/mL concentration in the FHD sample and the 40 mg/mL concentration in the OHD sample, which do not exhibit statistically significant differences (*p* > 0.05).

### 2.3. High-Performance Liquid Chromatography for the Individual Profiling of Polyphenols

The retention times, *m*/*z* signals and the concentrations of each compound identified are presented in Table 2.

The concentrations of epicatechin, rutin, and quercetin seem almost similar in the two samples studied. Differences between the samples were noted in resveratrol concentrations, higher for OHD (16.3 µg/mL) than FHD (11.57 µg/mL). Unlike flavonoids, the concentration of phenolic acids varied between the samples, the only similar concentrations being observed at rosmarinic (6.8 µg/mL) and β-resorcylic acid (25 µg/mL). Gallic, caffeic and ferulic acids were undetectable in the case of OHD, which was characterised by the presence of cumaric acid (4.11 µg/mL). Instead, cumaric acid was absent in the FHD sample, but other phenolic acids were detectable in different concentrations: gallic acid—1.67 µg/mL; caffeic acid—6.3 µg/mL; ferulic acid—12.66 µg/mL.

### 2.4. Antimicrobial Activity

**Figure 2:**
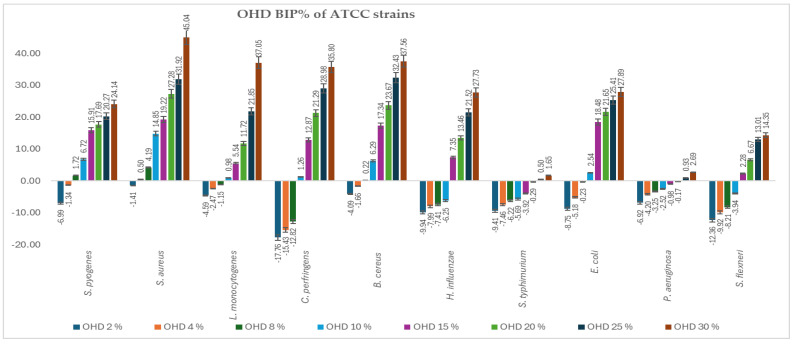
BIP% of OHD against the tested ATCC strains.

**Figure 3:**
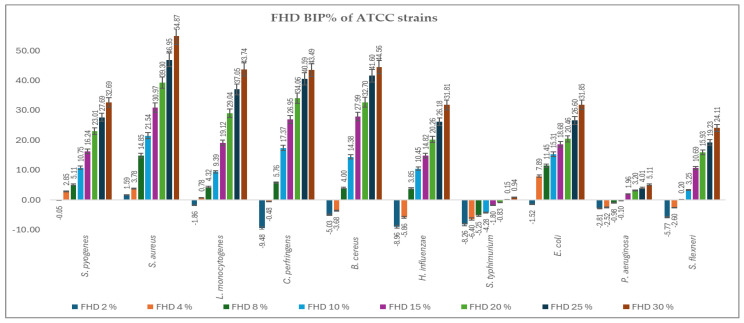
BIP% of FHD against the tested ATCC strains.

Figure 2 and Figure 3 present the bacterial inhibition percentage (BIP%) values of OHD (Figure 2) and FHD (Figure 3) against Gram-positive and Gram-negative ATCC bacteria, values calculated using Formulas (2) and (3) given in Section 4.7.

The comparative antimicrobial activity of OHD and FHD honeys showed that there was a difference in the inhibition capacity on a wide array of bacterial strains. The results suggest that FHD showed better antimicrobial activity compared to OHD concerning higher inhibitory values and lower MIC values.

*S. aureus* and *S. pyogenes* demonstrated greater sensitivity to both honeys, with notable inhibition observed at concentrations greater than 8%. FHD showed higher inhibition, with BIP% reaching 54.87% (30%) for *S. aureus* and 31.26% (30%) for *S. pyogenes*. *L. monocytogenes* exhibited a dose-dependent response, with FHD demonstrating significantly better efficacy than OHD, starting at 4%. FHD achieved a BIP% of 27.43% at 30%, indicating improved membrane penetration or compound synergy.

An interpretation concerning the type of bacteria involved, the most resilient Gram-positive strains were *B. cereus* and *C. perfringens*, which showed negative BIP% at low concentrations, especially for OHD. Only at 8% (5.76%) did FHD start to significantly inhibit *C. perfringens*, while at 30%, the inhibition increased to 43.96%. With *P. aeruginosa* and *S. flexneri* exhibiting low or negative BIP% at lower concentrations, indicating higher resistance because of their outer membrane barrier, the Gram-negative representatives showed a more variable and concentration-dependent trend. At ≥8 mg/mL, *E. coli* proved highly susceptible to both honeys, particularly FHD, which at 30% showed 31.85% inhibition. FHD significantly inhibited *H. influenzae*, with the BIP% increasing from 7.93% at 8% to 44.86% at 30%, suggesting that phenolic acids, such as ferulic and caffeic acid, which are abundant in FHD, may have synergistic activity. Although FHD showed more modest results, *S. typhimurium* responded moderately to both types of honey, clearly favouring OHD, which reached a maximum BIP% of 4.89%. *P. aeruginosa* exhibited a peak sensitivity of 2.41% (30%) to OHD. FHD’s low effectiveness against this strain was confirmed by its negligible or negative BIP%. All MIC values identified are presented in Table 3.

Comparing the two types of honey analysed, FHD inhibited most strains at a low concentration. MIC values ranged from around 2–8% for the most sensitive organisms (*S. aureus*, *S. pyogenes*, *E. coli, L. monocytogenes*, *H. influenzae*) as compared to 8–15% for the same strains when treated with OHD. Even at the highest concentration tested (30%), FHD showed higher BIP% in almost all species. Moreover, FHD displayed an inhibition percentage of 54.87 for *S. aureus* and 43.74 for *L. monocytogenes*, whereas OHD showed inhibition percentages of 45.04 and 37.05 for the same organisms, respectively. The superior performance of FHD is probably because it contains a considerably higher phenolic content than OHD, distributed across a wide range of phenolic compounds, including gallic acid, caffeic acid, and ferulic acid, among others, which are known to cause membrane-disruption and enzyme-inhibiting effects. These compounds could have a synergistic effect in potentiating the ability of honey to breach bacterial defences and inhibit growth.

In contrast, OHD showed activity against bacteria with a narrower spectrum, having positive effects only at higher concentrations. Under such conditions, it displayed selectivity; for instance, being capable of modest inhibition at a rate of 2.69% and 1.65% of *P. aeruginosa* and *S. typhimurium*, respectively, at 30%, its MIC values were much higher, at 25%, accounting for less potency. The moderate activity of OHD may be influenced by its specific flavonoid content, such as resveratrol, which may contribute selectively against certain resistant strains, but without broad-spectrum effectiveness.

In addition, both honeys had lower effects on such species that naturally resist them, including *S. typhimurium* and *P. aeruginosa*. This confirms much of the introductory literature on the resistance of these strains because of strong efflux and an impermeable outer membrane. Even in such cases, FHD still showed a slight superiority of activity, reinforcing the assertion of its better chemical composition.

### 2.5. Molecular Docking

**Figure 4:**
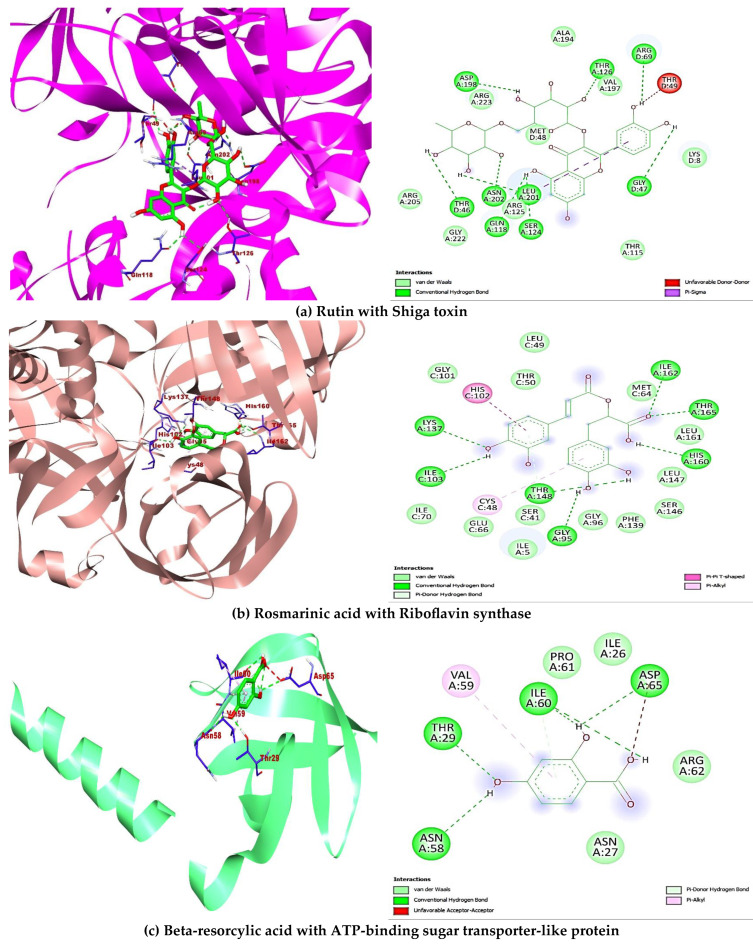

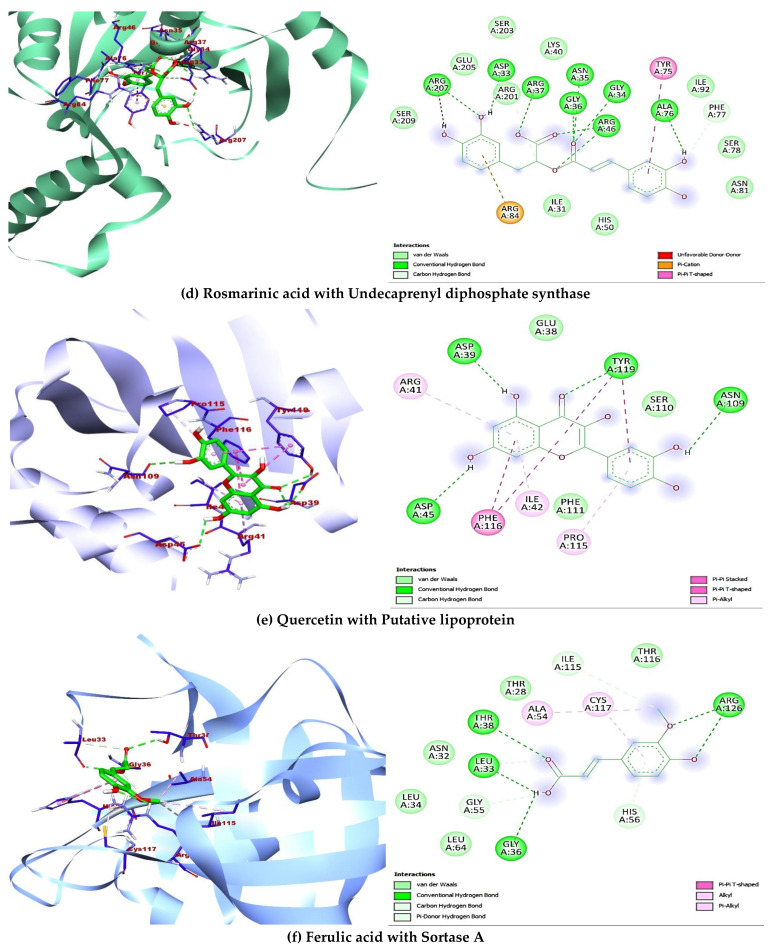

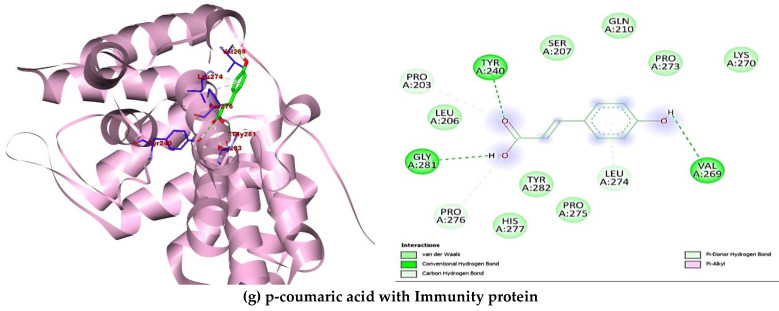
Showing 3-D and 2-D binding poses of the best docking outcome with each of the seven bacterial target proteins (**a**–**g**).

A total of ten (10) compounds identified by LC-MS from oak and fir honeydew honeys were successfully docked with seven (7) bacterial target proteins owing to their reported antimicrobial potential. Binding energies and active site binding amino acid residue interactions characterise the result. Thus, hydrophilic bonds (hydrogen bonds, carbon-hydrogen bonds), hydrophobic bonds (π, π-π, -alkyl, -sigma, -sulphur, -T-shaped), electrostatic bonds (π-anion/cation) were involved in the overall binding interactions and complex formation between the ligands (compounds) and receptors (proteins) (Table 4). Additionally, the docking scores revealed interesting binding energies within a concerted range measured in kcal/mol, 1dm0 (−9.1 to −5.9), 1i8d (−9.1 to −5.9), 2pp6 (−6.6 to −4.6), 4h8e (−9.4 to −5.8), 4r7r (−7.2 to −4.9), 5hu4 (−7.8 to −5.0) 5jkp (−8.7 to −5.6), respectively. Interestingly, rutin (−9.1 kcal/mol) gave the nine hydrophilic bonds with Shigella toxin protein (THR46, GLY47, ARG69, GLN118, SER124, THR126, ASP198, LEU201, ASN202), rosmarinic acid (−8.9 kcal/mol) showed seven hydrophilic bonds with riboflavin synthase (GLY95, ILE103, LYS137, THR148, HIS160, ILE162, THR165), beta-resorcylic acid (−4.8 kcal/mol) had four hydrophilic bonds (THR29, ASN58, ILE60, ASP65) with ATP-binding sugar transporter-like protein, again rosmarinic acid (−8.2 kcal/mol) fared better with undecaprenyl diphosphate synthase forming nine hydrogen bonds (ASP33, GLY34, ASN35, GLY36, ARG37, ARG46, ALA76, PHE77, ARG207207), quercetin (−7.0 kcal/mol) formed four hydrogen bonds (ASP39, ILE42, ASP45, ASN109, TYR119) with putative lipoprotein of *C. perfringens*, ferulic acid (−5.6 kcal/mol) had seven hydrogen bonds with *L. monocytogens* sortase A protein (LEU33, GLY36, THR38, GLY55, HIS56, ILE115, ARG126), finally p-coumaric acid showed six hydrogen bonds (PRO203, TYR240, VAL269, PRO276, GLY281) with immunity protein of *P. aeruginosa* (Figure 4). Other compounds interacted fairly with the target proteins and had good binding affinities.

### 2.6. ADME (Absorption, Distribution, Metabolism, and Excretion) Properties

The ADMET analysis performed on the six (6) bioactive compounds aimed to show any liabilities and possibly depict the safety pattern of utilising them in vivo. In particular, the compounds were assessed for cardiac toxicity, inhibition of cytochrome P450 isoforms, and hepatotoxicity (Table 5). Findings revealed that intestinal absorption was predicted to be moderate for beta-resorcylic acid, quercetin, ferulic acid, and p-coumaric acid. In contrast, rutin and rosmarinic acid had a low potential for intestinal absorption (23.4%, 32.5%), respectively. Additionally, except for quercetin, which showed a positive response to the CYP1A2 isoform, all the other bioactive compounds did not have inhibitory effects on any of the cytochrome proteins. The compounds also harmed hepatotoxicity and skin sensation predictors, although scoring for the blood–brain barrier was insufficiently distributed. More so, Rutin had the numerical violations for Lipinski, Ghose, Verber, Egan, and Muegge’s rules and had the lowest bioavailability score (0.17).

## 3. Discussion

Honeydew honey has gained popularity due to its unique nutritional, sensory, and various biological properties [58,59,60,61,62]. The biological properties are correlated with chemical composition variables, depending mainly on geographical origin, climate, and meteorological conditions, plant type, and insect species [63]. Generally, the results obtained in different studies mention a significant diversity of polyphenolic compounds, with varying values of TPC and TFC [58,64,65]. Can et al. demonstrated a concentration of TPC and TFC of 120.04 mg GAE/100 g and 3.10 mg QUE/100 g in Turkish oak honeydew honey [58]. Comparing three types of honeydew honey from Spain, Fernández-Estellé et al. observed that holm oak varieties had the highest TFC (1.78 mg QUE/g) and FC index (70 mg GAE/g honey), followed by forest and mountain honeydew honey [59]. Instead, Seijo et al. demonstrated that oak honeydew from the same country had a TPC of 134.8 mg GAE/100 g and TFC of 9.7 mg QUE/100 g [66]. In the present study, the values detected in Romanian OHD were higher, respectively, 701.37 mg GAE/kg and 395.18 mg QUE/kg. Similarly, slightly higher TPC and TFC were detected in FHD—844.50 mg GAE/kg and 498.01 mg QUE/kg. In contrast, Kuś et al. highlighted that Polish fir honeydew honey had a moderate total phenolic content (533.2 mg GAE/kg), while Jaśkiewicz et al. showed a concentration of 71.0 mg GAE/100 g, even though the plant origin of this honeydew from Poland is not specified [64,65].

A diversity of polyphenols was identified in different kinds of honeydew from Europe, such as salicylic acid, gallic acid, ferulic acid, coumaric acid, *p*-hydroxybenzoic acid, chlorogenic acid, vanillic acid, epicatechin, catechin, kaempferol, luteolin, pinocembrin, quercetin, rutin, and chrysin [67]. However, some phenolic compounds correlate with botanical origin. For example, myricetin and genistein were reported only in *Thymus vulgare* honeydew honey [68], coniferaldehyde, syringaldehyde, and hesperidin in *Mimosa scabrella* Bentham [69], and kynurenic acid in *Salix* spp. [70]. The present study highlighted that OHD and FHD contained similar flavonoids, rutin, epicatechin, and quercetin, in the same concentrations. Instead, Oroian et al., by studying five honeydews from the northeast part of Romania with unspecified botanical sources, remarked various concentrations of different flavonoids, such as myricetin (0–0.37 mg/100 g honey), chrysin (0–0.16 mg/100 g honey), pinocembrin (0.27–4.36 mg/100 g honey), quercetin (0.10–2.79 mg/100 g honey), apigenin (0–1.10 mg/100 g honey), kaempferol (0–0.60 mg/100 g honey), isorhamnetin (0–0.12 mg/100 g honey), luteolin (0–0.11 mg/100 g honey), and galangin (0.02–0.49 mg/100 g honey). Regarding the phenolic acids profile, the author found different concentrations of p-coumaric acid (0–4.35 mg/100 g honey), caffeic acid (0–1.92 mg/100 g honey), and gallic acid (0.02–0.26 mg/100 g honey) [71]. The present study showed that the main representative phenolic acid for both honeydew honeys studied was β-resorcylic (25.01 μg/mL). Also, rosmarinic acid was identified in both studied samples at a concentration of 6.8 μg/mL. Other phenolic acids, such as gallic acid, caffeic acid, coumaric acid, and ferulic acid, were the ones that made the difference between OHD and FHD. OHD contained only cumaric acid (4.11 μg/mL), while this compound was lacking in FHD, which was rich in ferulic acid (12.66 μg/mL) but also contained caffeic acid (6.3 μg/mL) and gallic acid (1.67 μg/mL). In contrast to our study, Hernandez et al. demonstrated that most of the Spanish oak honeydew honey tested contained, besides p-coumaric acid (mean = 0.594 mg 100 g^−1^), caffeic acid (mean = 0.176 mg 100 g^−1^), and also low concentrations of ferulic acid (mean = 0.071 mg 100 g^−1^). Like vanillic and chlorogenic acids, gallic acid was more infrequent in the samples [72]. Another study concluded that Polish honeydew is rich in trans-ferulic acid (221.29 µg/100 g), followed by p-coumaric (199.69 µg/100 g) and caffeic acid (143.5 µg/100 g), even though the botanical source is not specified [65].

The various phenolic compounds contribute to the diversity of the biological activities of honeydew honey due to their synergistic and antagonistic interactions. Generally, honey is recognised as an antioxidant natural remedy, capable of scavenging free radicals, reducing oxidative stress, and protecting against cell damage due to the content of resveratrol, epicatechin, rutin, quercetin, kaempferol, p-coumaric acid, caffeic acid, and ferulic acid [59,67,68]. Additionally, enzymes, amino acids, and carotenoids also enhance the antioxidant capacity [67]. Among different types of honey, honeydew honey stands out for its pronounced antioxidant activity, comparable to that of manuka honey [23,26]. Kačániová et al. demonstrated that forest honeydew from Slovakia exhibited antioxidant activity toward DPPH radicals in the 29.84–41.94% range [26]. In contrast, Jaśkiewicz et al. reported higher values for Polish honeydew honey, with a mean of 89.4% [59]. Moreover, the IC50 values for Turkish honeydew honey ranged from 12.56 to 76.20 mg/mL, depending on the botanical origin, with oak honeydew displaying the lowest IC50 (12.56 mg/mL) [52]. Greek oak honeydew appears to be more efficient, with an IC50 value of 7.14 mg/mL [69]. The current study identified an even lower IC50 value for oak honeydew, specifically 6.26 mg/mL. Higher antioxidant activity of FHD was observed compared to OHD, as the IC50 value was 5.16 mg/mL. This aspect is anticipated since the TPC value strongly correlates with antioxidant activity [59]. On the other hand, in addition to the synergistic effects of resveratrol and quercetin, known for their anti-inflammatory and antioxidant properties [70,71,72], FHD contains more phenolic acids, including caffeic acid, ferulic acid, and gallic acid, which contribute to enhanced antioxidant capacity [41,73,74,75].

Building on its chemical composition and antioxidant properties, honey also demonstrates remarkable antimicrobial activity. The ability to combat a wide range of pathogens has been well-documented in scientific studies [3,26,32,35,36,37,38]. However, the antimicrobial activity depends on different honey’s physicochemical properties and bacteria’s characteristics. Generally, Gram-positive bacteria are more susceptible to substances that target peptidoglycan than Gram-negative bacteria due to the lack of an outer lipid membrane [39]. Similarly to this hypothesis, the values of MIC were lower when studying the efficacy of the honey samples against Gram-positive bacteria, respectively, in the range of 2–10%, than against Gram-negative bacteria (4–25%). It is worth mentioning that FHD had lower MIC values than OHD for all strains studied except for *S. typhimurium*. Analysing the MIC values for each studied strain, it was observed that the most sensitive bacteria from the Gram-positive ones are *S. aureus*, with MIC values of 2% for FHD and 4% for OHD. For the Gram-negative ones, the lower MIC values were detected against *E. coli*, 4%, by testing FHD and 10% in the case of OHD, respectively. In contrast, fir honeydew honey from the Podkarpackie Province of Poland seems inefficient against *S. aureus* and *E. coli*, but also against *L. monocytogenes* [73]. This bacterial strain in our study was susceptible to both honeys studied. In contrast, Grabek-Lejko et al. claim that the MIC value of fir honeydew from the same country was 20.5 % for *S. aureus* [74]. Also, fir honeydew from Croatia is efficient against *S. aureus* and *S. epidermitis*, but only at a concentration of 0.25 g/mL [75]. The susceptibility of *S. pyogenes* to fir honeydew from other European countries was also demonstrated. For example, Dzugan et al. showed the efficacy of Polish fir honeydew honey against this bacterial strain at a concentration between 12.5% and 50% [73]. However, the current study highlighted that even lower concentrations of FHD are necessary to inhibit *S. pyogenes* growth, respectively, 4%, while the MIC value of OHD for the strain was 10%. Similarly, lower MIC was observed against *B. cereus* (8%) compared to the values reported in the literature [74].

The antimicrobial activity of honeydew honey studied against Gram-negative bacteria is also higher than that of honey from other countries [74,76]. Still, compared with another study from Bucovina, Romania, FHD and OHD are less efficient against *P. aeruginosa* and *S. typhimurium* [37]. Thus, Luca et al. sustained that the MIC values of Romanian honeydew honey are in the range of 3.15–12.50% for *P. aeruginosa* and 6.25– 12.50% for *S. typhimurium,* while in the current study, the MIC was 25% against both bacterial strains [37]. However, the author does not mention the botanical origin of honeydew honey. Moreover, the MIC value for *E. coli* found in the study was 6.25–12.5%, while the FHD from the present study seems to be more efficient against this bacterial strain, since the MIC found was 4% [37].

Given these observations, it is clear that Romanian OHD and FHD exhibit much higher total phenolic content (TPC) and total flavonoid content (TFC) compared to other European honeydew honey varieties, including those from Spain and Poland. Elevated phenolic levels are linked to greater antioxidant and antimicrobial properties in these honeys. This indicates that the unique environmental conditions in Romania may play a key role in shaping honeydew honey’s chemical characteristics and bioactive properties”.

In the attempt to understand and define the possible mechanism of inhibition to which the identified bioactive compounds act as therapeutic agents against certain bacteria of biological importance, impact on chemical alteration or disruption, and evasion of antibacterial resistance are standard mechanisms of inhibition [77]. The ten (10) compounds identified in the oak and fir honeydew honeys all depicted significant comparative binding affinity towards each of the bacterial target proteins. The binding poses, complex formation, strong hydrogen bonds, non-covalent bonds, and high binding energies recorded from each ligand-protein interaction are corroborated by the inhibitory potential exhibited by the bioactive compounds on the target proteins. Although six compounds (Figure 4) displayed better binding affinities and amino acid residue interactions (Table 4), rutin, rosmarinic acid, beta-resorcylic acid, quercetin [78], ferulic acid, and p-coumaric acid all suggested comprehensive potential in combating infections arising from foodborne bacterial infections [79].

The ADMET scores establish a thorough therapeutic framework for the bioactive compounds assessed, potentially enhancing their pharmacological effects. Importantly, their favourable bioavailability, human intestinal absorption, non-hepatotoxicity, and moderate clearance (Table 5) could enhance honey’s overall effectiveness against food-borne bacterial infections. Thus, the beneficial aspects of these in silico properties justify their consideration in calculating honey’s biological activity score. This further emphasises the role of the identified honey compounds in achieving the targeted antimicrobial effectiveness.

FHD exhibited stronger and broader-spectrum antibacterial activity, as reflected by lower MIC values for *S. aureus* (2%), *S. pyogenes* (4%), *L. monocytogenes* (4%), and *E. coli* (4%), as well as *H. influenzae* (8%). Its higher concentrations of ferulic, caffeic, and gallic acids, which have been shown in the literature to have bactericidal effects through membrane disruption, DNA damage, and inhibition of enzymatic targets like DNA gyrase and penicillin-binding proteins, are closely associated with this increased activity.

Additionally, the molecular docking studies supported the mechanism by which quercetin and ferulic acid inhibit bacteria, revealing their strong binding affinities to key bacterial enzymes. The therapeutic potential of these phenolic acids was emphasised further by their favourable ADME profiles, which featured high predicted gastrointestinal absorption and acceptable drug-likeness scores.

While FHD was less effective against *S. typhimurium* (MIC: 25%) and *P. aeruginosa* (MIC: 25%), OHD demonstrated significant selective efficacy against these two bacteria despite having lower antibacterial activity. This could be because it contains more resveratrol, a stilbenoid compound that inhibits biofilms, modulates the efflux pump, and inhibits quorum sensing in Gram-negative bacteria. This function is further supported by resveratrol’s molecular docking scores against membrane-associated targets. Resveratrol maintains its pharmacological significance despite having a lower oral bioavailability in its ADME profile because it can alter bacterial virulence pathways instead of directly causing cytotoxicity.

Both honeys demonstrated negative or insignificant BIP% values at lower concentrations (e.g., 4–6%), especially against *B. cereus, C. perfringens, S. flexneri*, and *P. aeruginosa*. This could reflect not only bacterial resistance mechanisms but also potential antagonistic interactions among honey constituents at subinhibitory doses. It is possible that certain phenolics or sugars may temporarily serve as nutrient sources for some bacteria when present below inhibitory thresholds.

The significant antimicrobial superiority of FHD against most tested strains indicates its potential as a natural antibacterial agent, primarily attributed to its polyphenol-rich composition and positive pharmacokinetic predictions. While OHD shows lower direct inhibition potency, it exhibits selective effectiveness against specific Gram-negative pathogens, suggesting that it may be worthy of further investigation for anti-virulence or supportive applications. These results emphasise the importance of combining phytochemical analysis, microbiological tests, and in silico modelling to better understand the therapeutic potential of complex natural products such as honeydew honeys.

## 4. Materials and Methods

### 4.1. The Preparation of Extracts

Two varieties of honeydew were taken into the study: oak honeydew honey (OHD) and fir honeydew honey (FHD). The samples were sourced from approved local producers in Sibiu (SC Apilife Ro SRL, Sibiu, Romania).

The choice of 70% ethanol was based on our previous studies [3], demonstrating its efficacy as a solvent for polyphenolic extraction from natural matrices, including honey and plant-based materials. Ethanol-water mixtures in the range of 60–80% are frequently reported to provide optimal extraction efficiency for a broad spectrum of phenolic compounds, balancing polarity to maximise both flavonoid and phenolic acid solubility.

Therefore, 1 g of each honey was mixed with 10 mL of 70% alcohol and vortexed for 15 min. The extraction was then continued on a hot plate stirrer (IDL, Freising, Germany) for 45 min at room temperature. After extraction, the samples were filtered and stored in a refrigerator at 4 °C for future analyses.

The extracts were filtered using 0.45 µm PTFE syringe filters (Sigma-Aldrich; Merck KGaA, Darmstadt, Germany), each with a diameter of 25 mm.

### 4.2. Chemicals

Sigma–Aldrich Chemie Gmbh (München, Germany) provided the reagents ethanol, Folin–Ciocalteu, gallic acid and quercetin standard, and 1,1-diphenyl-2-picrylhydrazyl (DPPH), while Geyer Gmbh (Renningen, Germany) provided the sodium carbonate, sodium nitrite, and aluminium chloride. For the chemical analysis, every reagent used was of analytical quality. Ascorbic acid was obtained from Lach-Ner Company (Neratovice, Czech Republic). All HPLC standards (Sigma-Aldrich, Merck KGaA, Darmstadt, Germany) and methanol (Merck KGaA, Darmstadt, Germany) were analytical-grade chemicals.

### 4.3. Determination of Total Phenolic Content (TPC)

The total polyphenol content was determined using the method described by Hulea et al. [3] with minor modifications. Briefly, 0.5 mL of each extract was mixed with 1.25 mL of Folin–Ciocalteu reagent and incubated at room temperature for 5 min; then 1 mL of 6% Na_2_CO_3_ was added. The mixture was vigorously stirred and incubated for 2 h at room temperature.

The absorbance values were measured at 750 nm using a Specord 205 UV-VIS spectrophotometer (Analytik Jena AG, Jena, Germany). The calibration curve was established using gallic acid as the standard, with the results expressed in mg GAE/Kg. Each sample was analysed in triplicate, and the results were reported as the mean ± standard deviation.

### 4.4. Determination of Total Flavonoid Content (TFC)

Our previous studies describe the method used to determine the total flavonoid content [4,79] with minor modifications as follows: a mixture of 1 mL of each extract, 0.3 mL of 10% AlCl_3_ and 0.3 mL of 5% NaNO_2_ was incubated at room temperature for 6 min. After this period, 2 mL of 1M NaOH and 6.4 mL of 70% ethanol were added. The mixture was vigorously shaken and incubated at room temperature for 30 min.

Absorbance values were measured at 415 nm using a Specord 205 UV-VIS spectrophotometer (Analytik Jena AG, Jena, Germany). The calibration curve was constructed using quercetin as the standard, with the measurement unit expressed as mg QUE/Kg.

Each sample was analysed in triplicate, and the results were reported as the mean ± standard deviation.

### 4.5. Individual Identification of Polyphenols by LC-MS Analysis

Determination of individual polyphenols by LC-MS analysis was performed using a Shimadzu chromatograph (Shimadzu 2010 EV, Kyoto, Japan) equipped with an SPD-10A UV detector, and MS detector (Shimadzu, Kyoto, Japan) EC 150/2 NUCLEODUR C18 Gravity SB 150 × 2 mm × 5 μm column (Macherey-Nagel Gmbh & Co., KG, Dueren, Germany).

Chromatographic conditions included mobile phases A—acidified water with formic acid (pH 3) (Merck KGaA, Darmstadt, Germany), and B—acetonitrile (Merck KGaA, Darmstadt, Germany) acidified with formic acid (pH 3). The gradient programme consisted of 0.01–20 min 5% B, 20.01–50 min 5–40% B, 40–55 min 40–95% B, and 55–60 min 95% B. The flow rate of solvent is 0.2 mL/min at a temperature of 20 °C. The wavelengths selected for monitoring were 280 nm and 320 nm. The calibration curves were made between 20 and 50 μg/mL. Calibration curves for each analysed compounds are as follows: f(x) = 6.04661e − 10^5^x − 3.04827 (gallic acid), f(x) = 7.36054e − 10^6^x + 3.55894 (caffeic acid), f(x) = 3.43873e − 10^5^x + 4.99711 (epicatechin), f(x) = 1.45563e − 10^5^x + 3.75512 (Beta − rezorcilic acid), f(x) = 4.34018e − 10^6^x + 3.48454 (cumaric acid), f(x) = 8.1887e − 10^6^x + 3.79724 (ferulic acid), f(x) = 2.21544e − 10^5^x + 1.3627 (rosmarinic acid), f(x) = 4.37174e − 10^5^x + 1.3537 (resveratrol), and f(x) = 1.3719e − 10^5^x + 3.97487 (quercetin). Analytical-grade chemicals were used for all reagents and solvents. Each sample was tested in triplicate. The MS signals and chromatograms are presented in the (Supplementary Material Figure S1).

### 4.6. DPPH Assay Antioxidant Capacity

The antioxidant activity was determined using the DPPH radical scavenging assay, following a standardised protocol adapted from previously reported methods [80,81,82] optimised for the characteristics of honey extracts. Briefly, to determine the free radical inhibition capacity, a series of dilutions were performed on the samples, obtaining the following final concentrations: 100 mg/mL, 40 mg/mL, 25 mg/mL, 16.67 mg/mL, and 12.5 mg/mL. A total of 1 mL of the diluted samples was mixed with 2.5 mL of 0.3 mM DPPH reagent, shaken, and left in the dark for 30 min. As a control, a sample was prepared in which the extract volume was replaced with 70% ethanol. In parallel, analyses were performed on five ascorbic acid samples (0.02–0.1 mg/mL), serving as a positive control. Absorbance values were measured at 518 nm using a Specord 205 UV-VIS (Analytik Jena AG, Jena, Germany) spectrophotometer. Each sample was analysed in triplicate, and the results were reported as the mean ± standard deviation. The results were obtained using the following formula:

(1) $$\mathrm{RSA}\;(\%)=(\frac{Ac-As}{Ac})\times 100$$

where A_c_ = absorbance value of the control sample,

A_s_ = absorbance value of the extract sample.

The IC_50_ value, representing the antioxidant capacity, was compared to that of ascorbic acid.

### 4.7. Antimicrobial Activity

The ATCC strains taken into study consisted of *S. pyogenes* (ATCC 19615), *S. aureus* (ATCC 25923), *L. monocytogenes* (ATCC 19114), C. *perfringens* (ATCC 13124), B. *cereus* (ATCC 10876), *H. influenzae type B* (ATCC 10211), *S. typhimurium* (ATCC 14028), *E. coli *(ATCC 25922), *P. aeruginosa* (ATCC 27853), and *S. flexneri* (ATCC 12022).

A 10^−3^ dilution (0.5 McFarland standard) of the fresh culture in Brain Heart Infusion (BHI) broth (Oxoid, CM1135) was used to test the antimicrobial activity of the honey extracts, as our previous study describes [79,83,84]. The extracts were added directly to the bacterial strain suspensions in different concentrations (2%, 4%, 8%, 10%, 15%, 20%, 25%, or 30%). The concentrations tested were chosen based on previous research and a literature review to cover a wide spectrum of concentrations and identify possible MIC values [3,4,32,35,37,38,83].

The MIC, defined as the lowest compound concentration that yields no visible microorganism growth, was determined by the measurement of optical density (OD) using the spectrophotometric method. The concentrations used to determine the minimum inhibitory concentration (MIC) were as follows: 2%, 4%, 8%, 10%, 15%, 20%, 25%, and 30% (*v*/*v*) of honey extract in Brain Heart Infusion (BHI) broth. These values were selected based on prior literature and preliminary screening to ensure an appropriate range that would capture the inhibition threshold for each bacterial strain.

Two indicators, BGP (bacterial growth percentage) and BIP (bacterial inhibition percentage), were calculated for interpreting the results, based on the following formulas:

(2) $$\mathrm{BGP}\;(\%)=\frac{OD\;sample}{OD\;negative\;control\;sample}\times 100$$

(3) BIP (%)=100−BGP(%)

where OD sample represents the optical density at 540 nm as a mean value of triplicate readings; OD negative control represents the optical density at 540 nm as a mean value of triplicate readings for the selected bacteria in BHI.

### 4.8. Molecular Docking

Molecular docking involving the ten (10) identified compounds by LCMS and seven (7) antimicrobial protein targets was performed with PyRx-virtual screening tool version 0.8 which had the AutoDock Vina algorithm and the empirical free energy scoring function. The 3d structure of the compounds was retrieved [85] in .sdf format and prepared as a ligand in .pdbqt saved format. The .pdb format of the bacterial target proteins (PDB ID: 1DM0, 1I8D, 2PP6, 4H8E, 4R7R, 5HU4, 5JKP) was downloaded from the protein data bank [86]. The proteins defined as macromolecules were all prepared for docking in .pdbqt format. The ligands were screened against all the proteins in a blind docking cubic grid coverage. The binding interactions between the ligands and the proteins were visualised in BIOVIA Discovery Studio v21.1.0.20298, while the binding affinities of the lowest energy score were collected in .csv format.

### 4.9. ADMET (Absorption, Distribution, Metabolism, Excretion, Toxicity) Properties

The drug-likeliness and pharmacokinetic properties of the hit compounds from the docking scores were subjected to the swissADME [87] prognosis to ascertain predictors of in vivo utilisation and for the pharmacokinetic attributes [88]. The canonical SMILES of the hits were recovered from PubChem [85].

### 4.10. Statistical Analysis

In case of the spectrophotometric analyses, the statistical analysis was performed using JASP version 0.19.3.0. A one-way ANOVA was applied to identify correlations between the evaluated parameters. Differences between the average values of the examined characteristics were tested for significance using the Tukey post hoc test, with a significance level set at *p* = 0.05.

## 5. Conclusions

This study offers an extensive analysis of Romanian oak (OHD) and fir (FHD) honeydew honeys, incorporating polyphenolic profiling, antibacterial efficacy testing, molecular docking, and ADME assessment. FHD exhibited notably higher total phenolic and flavonoid content, correlating with improved antioxidant and antibacterial properties. In vitro antimicrobial tests indicated a stronger inhibitory effect of FHD, especially against Gram-positive strains like *S. aureus*, *S. pyogenes*, and *L. monocytogenes*, alongside certain Gram-negative strains such as *E. coli* and *H. influenzae*. Fir honeydew honey exhibited superior and broader-spectrum antimicrobial activity, achieving lower MICs and higher BIP% values across most tested bacterial strains. In contrast, oak honeydew honey showed weaker and more variable activity, with limited efficacy, particularly against resistant Gram-negative strains such as *P. aeruginosa* and *S. typhimurium*. Molecular docking simulations reinforced the antibacterial results, with significant compounds like ferulic acid, quercetin, rosmarinic acid, and rutin showing strong binding affinities to bacterial target proteins linked to pathogenicity. ADME predictions affirmed the therapeutic advantages of several polyphenols, primarily ferulic acid and quercetin, exhibiting promising drug-likeness and absorption features. These results promote the pharmacological promise of honeydew honeys, notably FHD, as natural antimicrobial agents. The integration of chemical, biological, and computational techniques provides valuable insights into the mechanisms of their bioactivity, supporting further exploration for food preservation, pharmaceutical formulations, or complementary therapies for bacterial infections.

## Figures and Tables

**Table 1:** DPPH radical scavenging activity in the honey samples (% inhibition).

Concentration (mg/mL)	OHD	FHD	Concentration (mg/mL)	Ascorbic Acid
	Inhibition %			Inhibition %
12.5	5.48 ± 0.02 ^a^*	8.67 ± 0.03 ^b^	0.02	27.02
16.67	11.47 ± 0.04 ^c^	15.18 ± 0.01 ^d^	0.04	44.94
25	15.82 ± 0.02 ^e^	26.79 ± 0.40 ^f^	0.06	56.02
40	26.93 ± 0.02 ^f^	33.00 ± 0.02 ^g^	0.08	71.59
100	42.93 ± 0.01 ^h^	52.51 ± 0.01 ^i^	0.10	91.75
**IC50 (mg/mL)**	6.26 ± 0.30 ^C^	5.16 ± 0.22 ^B^		2.47 ± 0.09 ^A^

* Different lowercase letters (a–i) indicate statistically significant differences between samples according to one-way ANOVA followed by Tukey’s HSD test (*p* < 0.05). Means with the same letter are not significantly different. Different uppercase letters (A–C) indicate statistically significant differences between samples within the same row, as determined by one-way ANOVA followed by Tukey’s HSD test (*p* < 0.05). The IC_50_ for OHD was extrapolated by curve fitting, as the experimental values remained below 50% inhibition.

**Table 2:** The individual profile of polyphenols detected using the LC-MS method.

Individual Polyphenol Compounds			Concentration (µg/mL)	
Flavonoids	Ret. Time (min)	*m*/*z*	OHD	FHD
Epicatechin	6.70	289	7.39	7.42
Rutin	12.99	609	7.93	7.93
Quercetin	30.74	301	1.57	1.56
**Stilbenes**				
Resveratrol	26.62	227	16.3	11.57
**Phenolic acids**				
Gallic	3.68	169	nd	1.67
Caffeic	7.63	179	nd	6.3
Cumaric	12.27	163	4.11	nd
Ferulic	14.4	193	nd	12.66
Rosmarinic	23.93	359	6.81	6.8
β-Resorcylic	10.15	153	25.01	25

**Table 3:** MIC values (%) for OHD and FHD on selected Gram-positive and Gram-negative strains.

	*S.* *pyogenes*	*S.* *aureus*	*L. monocytogenes*	*C. perfringens*	*B.* *cereus*	*H. influenzae*	*S. typhimurium*	*E.* *coli*	*P. aeruginosa*	*S. flexneri*
**OHD** **(%)**	10	4	10	10	8	15	25	10	25	15
**FHD** **(%)**	4	2	4	8	8	8	25	4	15	8

**Table 4:** Molecular docking score between the ten identified compounds and seven bacterial target proteins.

Protein Targets (PDB ID)	Ligands	Pubchem CID	Binding Energy (kcal/mol)	Binding Interaction
Shiga toxin 1DM0	Beta-resorcylic acid	1491	−6.1	Hydrophilic bonds: LEU76, SER112, TYR114, ARG170; Hydrophobic bonds: VAL78, VAL162
	Caffeic acid	689043	−6.4	Hydrophilic bonds: VAL78, SER112, ARG170, THR200; Hydrophobic bonds: TYR114, VAL162
	Epicatechin	72276	−7.9	Hydrophilic bonds: VAL78, SER112, THR200, LEU201; Hydrophobic bonds: TYR114, VAL162, MET260; Electrostatic bonds: GLU167
	Ferulic acid	445858	−6.3	Hydrophilic bonds: TRP34, ASN35; Hydrophobic bonds: TRP34
	Gallic acid	370	−5.9	Hydrophilic bonds: ASP16, ASN32, THR54, GLY62, SER64; Hydrophobic bonds: NIL
	p-coumaric acid	637542	−6.2	Hydrophilic bonds: VAL78, SER112; Hydrophobic bonds: TYR114, VAL162
	Quercetin	5280343	−8.8	Hydrophilic bonds: VAL78, SER112, ARG170, THR200; Hydrophobic bonds: TYR114, VAL162
	Resveratrol	445154	−7.1	Hydrophilic bonds: NIL; Hydrophobic bonds: TRP34
	Rosmarinic acid	5281792	−7.8	Hydrophilic bonds: ASP18, ARG33, ASN35; Hydrophobic bonds: TRP34
	Rutin	5280805	−9.1	Hydrophilic bonds: THR46, GLY47, ARG69, GLN118, SER124, THR126, ASP198, LEU201, ASN202; Hydrophobic bonds: THR49, LEU201
Riboflavin synthase 1I8D	Beta-resorcylic acid	1491	−5.9	Hydrophilic bonds: GLY4, VAL6, THR50; Hydrophobic bonds: ILE5
	Caffeic acid	689043	−5.9	Hydrophilic bonds: THR3, VAL6, GLU93, ARG168; Hydrophobic bonds: ILE5, GLN7
	Epicatechin	72276	−9.1	Hydrophilic bonds: VAL6, SER146; Hydrophobic bonds: ILE5, ILE162
	Ferulic acid	445858	−6.8	Hydrophilic bonds: HIS102, THR148, THR165; Hydrophobic bonds: LEU147
	Gallic acid	370	−6.1	Hydrophilic bonds: VAL6, GLY39, THR50, SER146, ARG168; Hydrophobic bonds: ILE5
	p-coumaric acid	637542	−6.1	Hydrophilic bonds: HIS102; Hydrophobic bonds: THR165
	Quercetin	5280343	−7.6	Hydrophilic bonds: HIS31; Hydrophobic bonds: ILE5, ARG86
	Resveratrol	445154	−7.6	Hydrophilic bonds: CYS48; Hydrophobic bonds: ILE5, ILE162
	Rosmarinic acid	5281792	−8.9	Hydrophilic bonds: GLY95, ILE103, LYS137, THR148, HIS160, ILE162, THR165; Hydrophobic bonds: CYS48, HIS102
	Rutin	5280805	−9.1	Hydrophilic bonds: THR3, VAL6, GLN7, HIS31, GLU93, ARG168; Hydrophobic bonds: NIL
ATP-binding sugar transporter-like protein 2PP6	Beta-resorcylic acid	1491	−4.8	Hydrophilic bonds: THR29, ASN58, ILE60, ASP65; Hydrophobic bonds: VAL59
	Caffeic acid	689043	−5.0	Hydrophilic bonds: GLU18; Hydrophobic bonds: ALA14, ALA17, ILE32, PHE55
	Epicatechin	72276	−6.1	Hydrophilic bonds: ALA17, SER56; Hydrophobic bonds: ALA14, ILE32
	Ferulic acid	445858	−4.6	Hydrophilic bonds: ASP13, LEU39; Hydrophobic bonds: ILE16, PHE82, LYS85
	Gallic acid	370	−4.7	Hydrophilic bonds: ILE60, ARG62, ASP65; Hydrophobic bonds: NIL
	p-coumaric acid	637542	−4.8	Hydrophilic bonds: NIL; Hydrophobic bonds: ARG10, ALA14
	Quercetin	5280343	−6.6	Hydrophilic bonds: ARG10, ASP13, GLU18, GLY57; Hydrophobic bonds: ALA14, ALA17, ILE32
	Resveratrol	445154	−5.8	Hydrophilic bonds: ALA17, GLU18; Hydrophobic bonds: ASP13, ALA14, ILE32
	Rosmarinic acid	5281792	−6.2	Hydrophilic bonds: SER56, ASN83, GLY84, PRO86; Hydrophobic bonds: ASP13, ALA14, ALA17, LYS85
	Rutin	5280805	−6.6	Hydrophilic bonds: ARG10, SER56; Hydrophobic bonds: ASP13, ALA14, ALA17, ILE32
Undecaprenyl diphosphate synthase 4H8E	Beta-resorcylic acid	1491	−5.8	Hydrophilic bonds: PRO196, GLN215, TYR218, SER219; Hydrophobic bonds: TYR218; Electrostatic bonds: ASP195
	Caffeic acid	689043	−7.1	Hydrophilic bonds: HIS50; Hydrophobic bonds: ASN35, ALA76, PRO96, PHE99
	Epicatechin	72276	−7.8	Hydrophilic bonds: ASP33, ASN81, ARG84; Hydrophobic bonds: HIS50, ALA76, ILE92; Electrostatic bonds: ARG84
	Ferulic acid	445858	−6.6	Hydrophilic bonds: ALA76; Hydrophobic bonds: MET32, HIS50, LEU95
	Gallic acid	370	−6.3	Hydrophilic bonds: HIS50, ASN81, ARG84; Hydrophobic bonds: ASN35 Electrostatic bonds: ARG84
	p-coumaric acid	637542	−7.1	Hydrophilic bonds: HIS50; Hydrophobic bonds: ILE57, ALA76, PRO96, PHE99, PHE148
	Quercetin	5280343	−8.1	Hydrophilic bonds: MET32, ASP33, ASN35, HIS50, TYR75; Hydrophobic bonds: TYR75, ILE92; Electrostatic bonds: ARG84
	Resveratrol	445154	−8.6	Hydrophilic bonds: HIS50, ALA76; Hydrophobic bonds: ASN35, ILE92, PRO96, PHE99, PHE148
	Rosmarinic acid	5281792	−8.2	Hydrophilic bonds: ASP33, GLY34, ASN35, GLY36, ARG37, ARG46, ALA76, PHE77, ARG207207; Hydrophobic bonds: ARG207, TYR75; Electrostatic bonds: ARG84
	Rutin	5280805	−9.4	Hydrophilic bonds: GLY36, ARG37, ALA76, GLU80, ARG84, ARG201 Hydrophobic bonds: ARG46, SER78, ASN81; Electrostatic bonds: ARG84
Putative lipoprotein 4R7R	Beta-resorcylic acid	1491	−5.1	Hydrophilic bonds: ARG23, SER75, LYS77; Hydrophobic bonds: LYS77, TRP93
	Caffeic acid	689043	−5.6	Hydrophilic bonds: GLU38, ASP39, TYR119; Hydrophobic bonds: PHE116, TYR119
	Epicatechin	72276	−6.9	Hydrophilic bonds: GLU38; Hydrophobic bonds: TYR119; Electrostatic bonds: ASP39
	Ferulic acid	445858	−5.7	Hydrophilic bonds: ASP45, PRO115; Hydrophobic bonds: ARG41, ILE42, PHE111, PHE116, TYR119
	Gallic acid	370	−4.9	Hydrophilic bonds: ASP39, SER110; Hydrophobic bonds: PHE116, TYR119
	p-coumaric acid	637542	−5.5	Hydrophilic bonds: PRO115; Hydrophobic bonds: ILE42, PHE116
	Quercetin	5280343	−7.0	Hydrophilic bonds: ASP39, ASP45, ASN109, TYR119; Hydrophobic bonds: ARG41, ILE42, PRO115, PHE116
	Resveratrol	445154	−6.9	Hydrophilic bonds: ASP39; Hydrophobic bonds: ARG41, ILE42, TYR119
	Rosmarinic acid	5281792	−6.1	Hydrophilic bonds: GLU38, ASP39, ASP45, LYS112; Hydrophobic bonds: NIL; Electrostatic bonds: ASP39
	Rutin	5280805	−7.2	Hydrophilic bonds: GLU38, ASP39, PRO115; Hydrophobic bonds: ILE42, TYR119
Sortase A 5HU4	Beta-resorcylic acid	1491	−5.0	Hydrophilic bonds: GLY55, HIS56, CYS117; Hydrophobic bonds: ALA54, ARG126
	Caffeic acid	689043	−5.3	Hydrophilic bonds: LEU33, THR38, CYS117, ARG126; Hydrophobic bonds: ALA54
	Epicatechin	72276	−6.9	Hydrophilic bonds: HIS56, GLU98, CYS117, ARG126; Hydrophobic bonds: LEU33, ALA54; Electrostatic bonds: ARG126
	Ferulic acid	445858	−5.6	Hydrophilic bonds: LEU33, GLY36, THR38, GLY55, HIS56, ILE115, ARG126; Hydrophobic bonds: ALA54, CYS117
	Gallic acid	370	−5.5	Hydrophilic bonds: ASN29, GLY36, ALA54, HIS56; Hydrophobic bonds: THR28, LEU33, ALA54
	p-coumaric acid	637542	−5.4	Hydrophilic bonds: LEU33, THR38; Hydrophobic bonds: ALA54
	Quercetin	5280343	−7.0	Hydrophilic bonds: ILE115; Hydrophobic bonds: ALA54, VAL101, CYS117, ARG126
	Resveratrol	445154	−6.3	Hydrophilic bonds: HIS56, THR116; Hydrophobic bonds: ALA54, GLU100, ILE115, ARG126, CYS117
	Rosmarinic acid	5281792	−7.8	Hydrophilic bonds: GLY36, ALA54, GLU98, GLU100, THR116, CYS117; Hydrophobic bonds: THR28, LEU33, ILE115, ARG126
	Rutin	5280805	−7.0	Hydrophilic bonds: HIS56, GLU100, ILE115; Hydrophobic bonds: ALA54, VAL101, CYS117, ARG126
Immunity protein 5JKP	Beta-resorcylic acid	1491	−5.7	Hydrophilic bonds: PRO203, TYR240; Hydrophobic bonds: LEU274, TYR282
	Caffeic acid	689043	−5.7	Hydrophilic bonds: TYR240, VAL269, LEU274, GLY281; Hydrophobic bonds: PRO273
	Epicatechin	72276	−6.6	Hydrophilic bonds: GLN117, PRO261; Hydrophobic bonds: PRO152, ALA185; Electrostatic bonds: ARG264
	Ferulic acid	445858	−5.6	Hydrophilic bonds: GLN210, TYR240, GLY281; Hydrophobic bonds: VAL269, LEU274, HIS277
	Gallic acid	370	−6.0	Hydrophilic bonds: TYR240, LEU274, GLY281, TYR282; Hydrophobic bonds: LEU274, TYR282
	p-coumaric acid	637542	−5.5	Hydrophilic bonds: PRO203, TYR240, VAL269, PRO276, GLY281; Hydrophobic bonds: NIL
	Quercetin	5280343	−6.9	Hydrophilic bonds: LYS55, ALA259, ASP269; Hydrophobic bonds: ALA51, ALA54, LYS55, PRO261
	Resveratrol	445154	−6.8	Hydrophilic bonds: LEU274; Hydrophobic bonds: TYR282, TRP289
	Rosmarinic acid	5281792	−6.6	Hydrophilic bonds: LYS55, ARG60, ASN83; Hydrophobic bonds: ALA54, TRP62, LEU87, PRO261
	Rutin	5280805	−8.7	Hydrophilic bonds: HIS82, GLN215, ASP262, ARG264; Hydrophobic bonds: TYR184

The overall binding interactions observed between the ligands and protein targets classically indicate how some of the key amino acid residues of the proteins were bonded with each of the identified compounds. Table 4 defines the amino acid residues potentially involved in the hydrogen bond formation and strong hydrophobic interactions. Particularly, the shiga toxin TYR114 [54], riboflavin synthase THR148, HIS102, CYS48 [55], sortase A CYS117, ARG126 [56], while immunity protein amino acid residues TYR283, TRP289 [57], served as gatekeeper residues of these respective proteins and participated wholly in the binding interactions of the ligand-protein complexes. The amine moeity, pyrimidine ring, hydroxyl, and carbonyl backbone of the amino acid residues were frequently involved in both the hydrophylic and hydrophobic interactions.

**Table 5:** ADMET Predictors of Pharmacokinetic parameters for the six best docked compounds.

Parameters	Rutin	Rosmarinic Acid	Beta-Resorcylic Acid	Quercetin	Ferulic Acid	p-Coumaric Acid	Unit
**Absorption**							
Water solubility	−2.892	−3.059	−2.183	−2.925	−2.817	−2.37	Numeric (log mol/L)
CaCO_2_ permeability	−0.949	−0.937	0.653	−0.229	0.176	1.61	Numeric (log Papp in 10^−6^ cm/s)
Intestinal absorption (human)	23.446	32.516	70.99	77.207	93.685	88.401	Numeric (% Absorbed)
Skin permeability	−2.735	−2.735	−2.735	−2.735	−2.72	−2.735	Numeric (log Kp)
P-glycoprotein substrate	Yes	Yes	No	Yes	No	No	Categorical (Yes/No)
P-glycoprotein I inhibitor	No	No	No	No	No	No	Categorical (Yes/No)
P-glycoprotein II inhibitor	No	No	No	No	No	No	Categorical (Yes/No)
**Distribution**							
VDss (human)	1.663	0.393	−1.816	1.559	−1.367	0.011	Numeric (log L/kg)
Fraction unbound (human)	0.187	0.348	0.651	0.206	0.343	0.751	Numeric (Fu)
BBB permeability	−1.899	−1.378	−0.757	−1.098	−0.239	0.181	Numeric (log BB)
CNS permeability	−5.178	−3.347	−3.317	−3.065	−2.612	−3.24	Numeric (log PS)
**Metabolism**							
CYP2D6 substrate	No	No	No	No	No	No	Categorical (Yes/No)
CYP3A4 substrate	No	No	No	No	No	No	Categorical (Yes/No)
CYP1A2 inhibitor	No	No	No	Yes	No	No	Categorical (Yes/No)
CYP2C19 inhibitor	No	No	No	No	No	No	Categorical (Yes/No)
CYP2C9 inhibitor	No	No	No	No	No	No	Categorical (Yes/No)
CYP2D6 inhibitor	No	No	No	No	No	No	Categorical (Yes/No)
CYP3A4 inhibitor	No	No	No	No	No	No	Categorical (Yes/No)
**Excretion**							
Total Clearance	−0.369	0.25	0.603	0.407	0.623	−6.797	Numeric (log mL/min/kg)
Renal OCT2 substrate	No	No	No	No	No	No	Categorical (Yes/No)
**Toxicity**							
AMES toxicity	No	No	No	No	No	Yes	Categorical (Yes/No)
Maximum tolerated dose (human)	0.452	0.152	0.89	0.499	1.082	0.593	Numeric (log mg/kg/day)
hERG I inhibitor	No	No	No	No	No	No	Categorical (Yes/No)
hERG II inhibitor	Yes	No	No	No	No	No	Categorical (Yes/No)
Oral Rat Acute Toxicity (LD_50_)	2.491	2.811	2.284	2.471	2.282	2.482	Numeric (mol/kg)
Oral Rat Chronic Toxicity (LOAEL)	3.673	2.907	1.857	2.612	2.065	4.375	Numeric (log mg/kg bw/day)
Hepatoxicity	No	No	No	No	No	No	Categorical (Yes/No)
Skin Sensitisation	No	No	No	No	No	No	Categorical (Yes/No)
*Tetrahymena Pyriformis* toxicity	0.285	0.302	0.285	0.288	0.271	0.285	Numeric (log ug/L)
Minnow toxicity	7.677	2.698	2.734	3.721	1.825	5.76	Numeric (log mM)
**Oral bioavailability**							
Lipinski’s rules	3	0	0	0	0	0	Violation (Numeric)
Ghose rules	4		3				Violation (Numeric)
Verber’s rules	1	1					Violation (Numeric)
Egan’s rules	1	1					Violation (Numeric)
Muegge rules	1		1		1	1	Violation (Numeric)
Bioavailability score	0.17	0.56	0.56	0.55	0.85	0.85	

Papp: Apparent permeability, BBB: blood–brain barrier, CYP: cytochrome P450, OCT2: Organic cation transporter 2, hERG: human ether-a-go-go related gene, LOAEL: lowest observed adverse effect level, LD_50_: 50% lethal dose.

## Data Availability

The raw data supporting the conclusions of this article will be made available by the authors on request.

## Supplementary Materials

The following supporting information can be downloaded at https://www.mdpi.com/article/10.3390/antibiotics14060592/s1, Figure S1: A. Chromatogram and *m*/*z* signal for FHD sample; B. Chromatogram and *m*/*z* signal for OHD sample.
