# Supplementary material for: Integrated Assessment of Antibacterial Activity, Polyphenol Composition, Molecular Docking, and ADME Properties of Romanian Oak and Fir Honeydew Honeys
Source: Antibiotics (Basel). 2025 Jun 8;14(6):592. doi: 10.3390/antibiotics14060592 (PMC12189776; doi:10.3390/antibiotics14060592)
Supplement: Supplementary file 1 [file antibiotics-14-00592-s001.zip › antibiotics-3665706-supplementary.pdf]

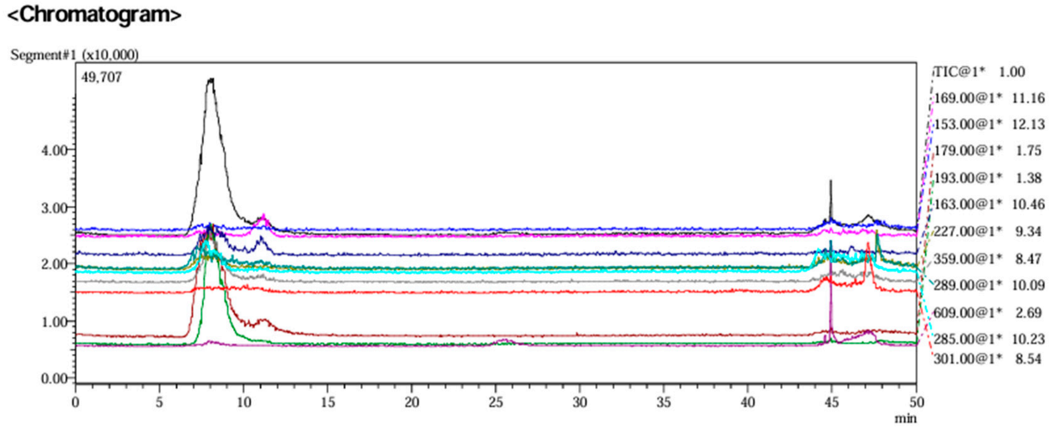

A

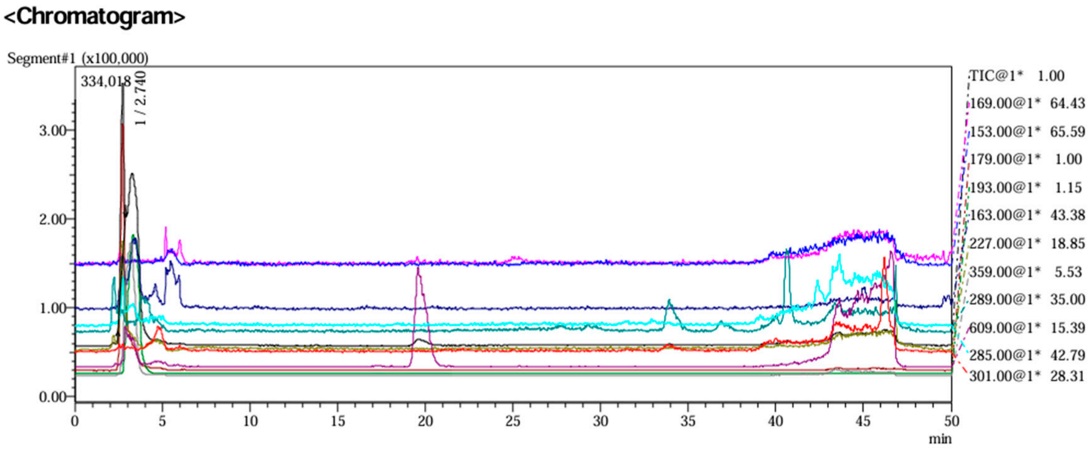

B

Figure S1: A. Chromatogram and m/z signal for FHD sample; B. Chromatogram and m/z signal for OHD sample
